# Dietary diversity, eating habits, dietary patterns, food choice, and associated factors among adolescent girls: a convergent parallel mixed-method study in the Mion District of Ghana

**DOI:** 10.1017/jns.2025.17

**Published:** 2025-03-26

**Authors:** Raihana Al-Hassan, Mavis A. Ayimbire, Ambrose Atosona, Humphrey Garti, Anthony Wemakor, Inge D. Brouwer, Fusta Azupogo

**Affiliations:** 1 Tamale Teaching Hospital, Department of Public Health, Nutrition Unit, Tamale, Ghana; 2 Department of Nutritional Sciences, School of Allied Health Sciences, University for Development Studies, Tamale, Ghana; 3 Department of Child Health, Tamale Teaching Hospital, Tamale, Ghana; 4 Directorate of Child Health, Komfo Anoakye Teaching Hospital, Kumasi, Ghana; 5 Africa Centre for Health Research and Development, Tamale, Ghana; 6 Department of Biochemistry and Biotechnology, College of Science, Kwame Nkrumah University of Science and Technology, Kumasi, Ghana; 7 Division of Human Nutrition and Health, Wageningen University and Research, Wageningen, The Netherlands; 8 International Food Policy Research Institute (IFPRI), Washington, DC, USA; 9 Department of Family and Consumer Sciences, Faculty of Agriculture, Food and Consumer Sciences, University for Development Studies, Tamale, Ghana; 10 Institute for Global Nutrition and Department of Nutrition, University of California, Davis, USA

**Keywords:** Adolescent girls, Dietary diversity, Dietary patterns, Eating habits, Food choice, Ghana, ASFs, Animal Source Foods, DDS, Dietary Diversity Score, FIES, Food Insecurity Experience Scale, F&V, Fruits and Vegetables, LMICs, Lower- middle-Income Countries, MDD-W, Minimum Dietary Diversity for Women, SES, Socio-economic Status, T.Z, Tuo Zaafi

## Abstract

Understanding adolescents’ diet and eating behaviours is crucial for informing public health strategies and policies, allowing interventions to be tailored effectively to enhance dietary patterns and improve overall health and quality of life. This study examined dietary patterns, dietary diversity, eating habits, food choice and the factors influencing these among adolescent girls in the Mion District, Ghana. A convergent parallel mixed-method design employing in-depth interviews (*n* = 30), two focus group discussions *(n = 10)* and survey data from 882 mother-daughter pairs was used. Key findings indicate that approximately 90% of girls achieved the minimum dietary diversity for women of reproductive age (MDD-W), with most consuming three meals daily. Staples were eaten daily, while fruits and unhealthy foods were less frequently eaten. Moderate/severe food insecurity was negatively associated with the MDD-W, whereas larger household size was positively correlated with the MDD-W. Older girls were less likely to eat breakfast, while household crop farm diversity increased the odds of eating supper on weekends. Moderate/severe food insecurity was inversely associated with intake of animal-sourced foods (ASFs), fruit, and unhealthy foods but positively correlated with vegetable consumption in the past month. Maternal non-literacy was linked to a lower intake of unhealthy foods, while specific ethnic backgrounds negatively influenced ASF intake. Wealthier households demonstrated higher consumption of staples. Qualitatively, health considerations, availability, taste, and cravings were major influencers of food choices. Food aversions were often tied to intolerance, fatigue from consuming the same foods repeatedly, and preferences related to taste and texture. These findings underscore the need for targeted nutritional interventions considering socio-economic and household factors to improve adolescent girls’ dietary habits and overall health.

## Background

Adolescence is a time in human growth and development that follows childhood but precedes adulthood and is characterised by physiological, psychosocial, and cognitive development.^(1,2)^ Alongside this pubertal stage comes a growth spurt that increases the need for macro and micronutrients.^(2)^

The increase in energy and nutrient needs during this time is accompanied by additional socioenvironmental elements that influence adolescents’ dietary preferences and nutrient intake and, consequently, their nutritional status.^(3)^ Adolescents’ eating patterns and behaviours are influenced by various factors, including peer influences, parental modelling, food availability, food preferences, costs, convenience, personal and cultural beliefs, and the mass media.^(1,4)^ Although many factors can affect an adolescent’s eating habits and food intake, research indicates that food parenting practices are the most significant in the food consumption behaviours of adolescents. Adolescent knowledge, attitudes and practices on healthy eating are influenced by parental knowledge, intake, attitudes, parenting style, time and the availability and accessibility of food in the home.^(5)^

Micronutrient deficiencies, specifically iron deficiency resulting from inadequate nutrient intake are a serious public health problem among adolescent girls globally.^(1)^ For instance, a high prevalence of iron deficiency anaemia among rural adolescent girls (64.6%) has been reported, especially from the northern savannah agro-ecological zone of Ghana.^(6)^ Girls are more vulnerable to nutritional problems compared to boys because of the biological differences among them during puberty (e.g., blood loss related to menstruation).^(7)^ Further, gender discrimination in the family’s food distribution and access puts teenage girls at a heightened risk of malnutrition.^(8)^ Adolescent girls are an important target group for nutrition and health interventions, as their nutritional status and health, both before and during pregnancy, significantly impact their well-being and can lead to intergenerational transmission of health (dis)advantages to their offspring.^(7,9,10)^ Ensuring adequate nutrition during this critical period can improve maternal health, enhance birth outcomes, and help break cycles of malnutrition and poverty, thus promoting long-term health for both mothers and their children.

Improved dietary diversity has been shown to improve the micronutrient adequacy of diet in different population groups.^(11)^ The minimum dietary diversity for women (MDD-W) indicates food-group diversity that captures micronutrient adequacy, a crucial aspect of diet quality. MDD-W is a dichotomous indicator of whether women aged 15 to 49 years have consumed at least five of ten defined food groups the previous day or night.^(12)^

School-age children and adolescents in low-and-middle-income countries, such as Ghana, have been found to consume suboptimal diets.^(13)^ Meal skipping, unhealthy eating patterns, and inadequate dietary diversity have been reported among adolescents in urban settings of Ghana, indicating that food consumption patterns are evolving.^(14–16)^ For instance, it has been noted that adolescents in Ghana primarily consume sugar-sweetened beverages, calorie-dense and low-nutrient snacks.^(13,14,17)^ However, little is known about rural adolescent girls’ dietary behaviours and practices in Ghana.

Insights into adolescent girls’ eating behaviours can guide public health strategies and policies such as school-based nutrition programmes, educational campaigns, and policies targeting food marketing to young people. By comprehensively understanding why adolescent girls eat what they eat, interventions can be better tailored to improve their dietary patterns and, consequently, their overall health and quality of life. In this study, we employed a mixed-methods approach to investigate the factors influencing MDD-W, eating habits, and dietary patterns among adolescent girls in rural Northern Ghana. We also explored the motivations behind their food choices. By identifying specific dietary habits and the underlying reasons for these choices, this study can help tailor nutritional interventions to the needs and preferences of adolescent girls in Northern Ghana. This is particularly important in settings where non-specific interventions may not address the unique barriers and facilitators to healthy eating, thereby increasing the effectiveness of programmes to enhance their health outcomes.

Specific research questions include the following:

1. What is the dietary diversity of adolescent girls in the Mion District?
2. What are the eating habits and dietary patterns of adolescent girls in the Mion District?
3. What are the factors associated with dietary diversity, eating habits and dietary patterns of adolescent girls in the Moin district?
4. What reasons do adolescent girls have for eating the foods they eat?

We therefore hypothesise that the eating habits, dietary diversity, dietary pattern, and food choices of adolescent girls can be influenced by multiple factors operating across the socio-ecological framework.

## Methods

### Study design

This study utilises data from the extensive survey of the Ten2Twenty-Ghana study,^(18)^ which was conducted in November/December 2018. The Ten2Twenty-Ghana study utilises a convergent parallel mixed-method approach with cross-sectional quantitative data and qualitative data from in-depth interviews and focus group discussions. It began with a thorough cross-sectional survey (*n* = 1057) and, 2 months later, a follow-up double-blind, randomised control trial (*n* = 621). Qualitative in-depth interviews and focus groups were also conducted at baseline on several topics regarding dietary intake, reproductive and sexual health, and psychosocial health. This study was conducted according to the guidelines laid down in the Declaration of Helsinki, and all procedures involving human subjects/patients were approved by the Navrongo Health Research Centre Institutional Review Board (NHRCIRB323). Written informed consent was obtained from all girls and their parents.

### Study area

The Ten2Twenty study was conducted in the Mion District in the Northern region of Ghana. The district is situated between latitudes 90-35 north, 00-30 west, and 00-15 east in Ghana’s Northern Region, in the eastern corridor. Sang, the biggest community in the district, serves as its capital. The area experiences two seasons with a typical tropical climate: a dry season from November to March and a wet season from April to October. Based on the 2021 Ghana Population and Housing Census, Mion District has an estimated 94,930 residents, most of whom (89.6%) reside in rural areas. The district has a high percentage of illiteracy (77.7%), and the people’s primary livelihood is agriculture.^(19)^ Data on nutrition and health in the district are scanty as the district is relatively new, carved out of the Yendi Municipal Assembly in 2012. However, the main crops cultivated include cereals and root tubers, often consumed as staples.

Our earlier analysis shows that 12–50% of adolescent girls suffered from iron deficiency, depending on the biomarker used (plasma ferritin or transferrin receptor concentrations). Additionally, approximately one-quarter were identified with iron deficiency anaemia, with anaemia prevalence being severe.^(20)^ Additionally, around 20% of our sample population reported depressive symptoms, highlighting the multifaceted health challenges faced by this population.^(21)^

### Study population and population for analysis

**Figure 1.**
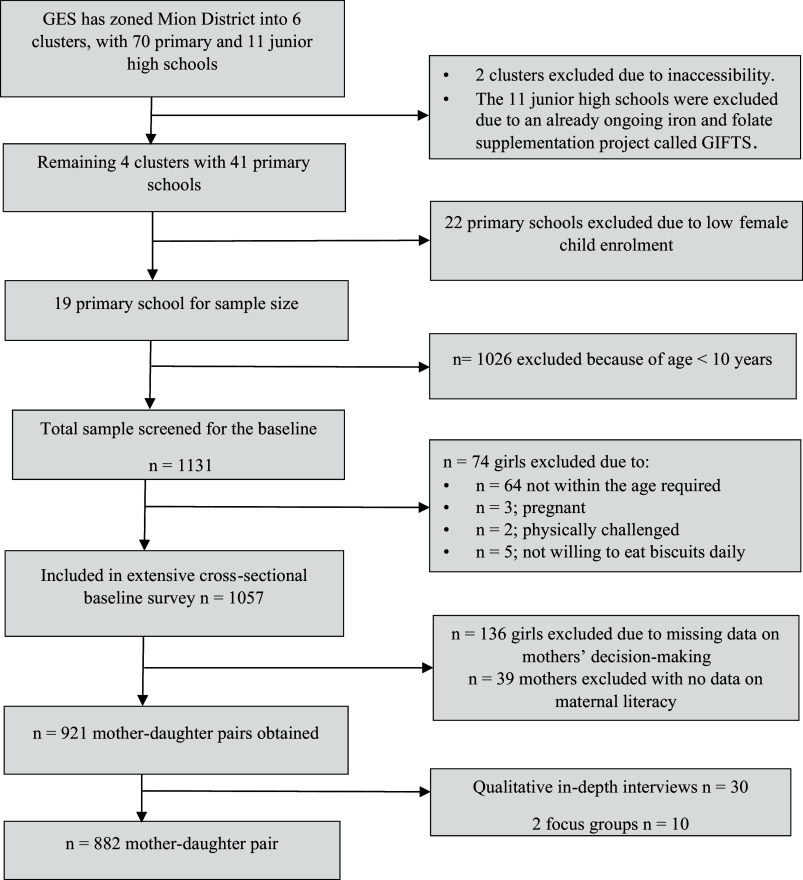
Flowchart for the selection of the study population.

The study’s target population consisted of pre-menarche and post-menarche teenage girls between the ages of 10 and 17 years, seemingly healthy, non-pregnant, nonlactating, and their mothers who lived in the Mion District in Ghana’s Northern region. Girls were conveniently chosen from nineteen basic schools throughout the Mion district, using secondary enrolment statistics provided by the Ghana Education Service. The selection process involved four educational circuits (clusters), incorporating all four urban schools in Sang and fifteen larger rural schools. Participants were screened using a 16-item questionnaire that assessed various factors, including menarche status, pregnancy and lactation status, health condition, medication use, iron supplement consumption, and prior participation in research related to drugs, supplements, or food. Girls who met the inclusion criteria were subsequently invited to participate in the study along with their mothers. Finally, a sample of 882 mother-daughter pairs was utilised for this study, accounting for missing data related to mothers’ decision-making and maternal literacy, which affected 175 pairs (Fig. 1).

The qualitative data was limited to only the adolescent girls. A random sample of 30 girls, including at least one girl from each participating school, was chosen for the qualitative in-depth interviews. Two of the four clusters in the Ten2Twenty-Ghana study were randomly selected, and one school was randomly chosen from each cluster for a focus group discussion consisting of 5 members in each group.

### Data collection procedure

A convergent parallel mixed-method approach was used to collect the data, combining quantitative and qualitative techniques. The quantitative data collection tools included anthropometry and one-on-one interviews, including a qualitative 24-hour recall and a one-month food frequency questionnaire, while the qualitative data collection method included in-depth interviews and focus group discussions. In November 2017, a pilot survey was conducted in the nearby Yendi municipality to pre-test the questionnaire.

### Outcome variables

Dietary diversity score, a dependent variable for this study, was evaluated by a qualitative 24-hour dietary recall (24hR), utilising the 10-food indicator for women of reproductive age.^(12)^ As part of the 24-hour recall, the girls were first requested to list everything they had eaten and drunk the day before, inside and outside the house (including at school). The 10-food-group indicator used included staples (grains, white roots, tubers, and plantains) (1), pulses (beans, peas, and lentils) (2), nuts and seeds (3), dairy (4), meat, poultry and fish (5), eggs (6), dark green leafy vegetables (7), other vitamin A-rich fruits and vegetables (8), other vegetables (9), and other fruits (10). A girl received a score of ‘1’ if she ate at least one food item from the food category and a score of ‘0’ if she did not. The DDS was created by summing the scores for each food group, with a minimum achievable score of 0 and a maximum score of 10. Girls who had a DDS of 5 or more met the minimum dietary diversity for women of reproductive age (MDD-W), and girls who had a DDS of less than 5 failed to meet the MDD-W.^(12)^

Additionally, the 10-food-group indicator^(12)^ was used to assess the girls’ dietary patterns in the last 30 days with a 1-month food frequency questionnaire ranging from 0 to 30. The girls were asked how frequently they ate food items from each of the food groups in the last month with frequency options ranging from ‘never’, ‘once per month’, ‘once per week’, ‘2-4 times weekly’, ‘5-6 times weekly’, to ‘once daily’. Each frequency was assigned a continuous score based on the number of days: ‘never’ was scored as 0, ‘once per month’ as 1, ‘once per week’ as 4, ‘2-4 times weekly’ as 12, ‘5-6 times weekly’ as 22, and ‘once daily’ as 30. In addition to examining fruit and vegetables (F&V) separately as outcome variables, we also analysed the combined measure by calculating the average frequency of consuming F&V. We calculated the mean frequency of consumption for both animal-source foods (ASFs), including dairy, meat, eggs, and fish and unhealthy foods, such as sweets, sugar-sweetened drinks, and savoury fried snacks, as outcome variables.

Lastly, the eating habits of the girls were assessed through six questions, which included how often the girls consumed the following: (1) breakfast during weekdays, (2) breakfast during weekends, (3) lunch during weekdays, (4) lunch during weekends, (5) supper during weekdays, (6) supper during weekends. Based on the data distribution, we computed binary outcome variables for breakfast, lunch and dinner being eaten ≤ 3 days and > 3 days for the weekday eating habits. Eating habits for weekends were re-grouped as either one of the weekend days or both weekend days. Likewise, binary outcome variables were defined for eating all three meals on weekdays and weekends.

### In-depth interviews and focus group discussion

In-depth interviews are the best method for gathering information about people’s backgrounds, viewpoints, and experiences.^(22,23)^ Hence, the approach was used to obtain details of eating patterns and the justifications for following a particular consumption pattern. Again, the focus group discussions included information on the girls’ knowledge, attitudes, and practices concerning their dietary habits. Two skilled research assistants led the focus group interviews; one facilitated the conversations while the other took notes and digitally recorded the sessions. The discussions were transcribed deductively using a pre-designed format.

### Demographic and socio-economic related covariates

#### Girl-level variables

Individual attributes of the girls: The girls’ characteristics including their ethnicity and religion, were classified as categorical variables; age, entered as a continuous variable; and menarche status, was dichotomous (pre- or post-menarche).

#### Mother-level variables

Maternal factors included literacy (dichotomous) and decision-making autonomy. Maternal decision-making autonomy was based on the Demographic and Health Survey 8-item final decision-making index.^(24)^ For each of the 8 items, a score of 1 was assigned to each instance where a mother participated in decision-making independently or with someone else in the household. Conversely, a score of 0 was given if she was not involved in decision-making. These scores were summed, with potential scores ranging from 0 to 8. A higher cumulative score indicated greater involvement in household decision-making.

### Household-level variables

#### Household wealth index

The International Wealth Index, which has a minimum score of 25 and a maximum score of 100^(25)^ was employed to determine the household’s socio-economic status (SES). The International Wealth Index rates households according to their possession of durable goods like a TV, refrigerator, phone, bicycle, car, and household items categorised as inexpensive (under $300), as well as the availability of sanitary facilities, the quality of water, and electricity, and the type of flooring they have.

#### Household food security

The Food Insecurity Experience Scale (FIES) was employed to determine the girls’ household food security status.^(26)^ The scale consists of eight yes/no questions, from mild food insecurity (questions 1–3) to severe food insecurity (questions 7–8). A yes response earns a score of 1, while a no response receives a score of 0. The eight items’ scores were summed to create the FIES score, ranging from 0 to 8. The FIES score was then divided into four groups: 0 to 2 represented food security, 3 to 4 represented mild food insecurity, 4 to 6 represented moderate food insecurity, and 7 to 8 represented severe food insecurity.

#### Household rooster

A household rooster captured data on parental education (categorical), occupation (categorical), sex (categorical), and literacy (dichotomous). Further, household size, female-to-male ratio, and literacy ratios were computed from the household roster following the guidelines of the Ghana Statistical Service.^(19,27)^ The household roster also included data on paternal education, literacy, and occupation, but these data were dropped from the analysis because of homogeneity.

#### Agriculture biodiversity of household

Farm diversity of household captured data on the different food crops households cultivated in the last farming season and the different types of farm animals and poultry/birds that households owned for the past year. The crop species included cereals and grains (1), root tubers (2), legumes, nuts, and seeds (3), vegetables (4), and fruits (5). Animal species included ruminants (1), poultry and birds (2). If the household cultivated or owned at least one of the food crops or farm animals, they received a score of ‘1’, otherwise a score of ‘0’. We created a variable for crop and animal diversity, a simple count of the number of different groups/types of farm crops or animals. The crop diversity varied from 0 to 5, while the animal diversity ranged from 0-2. Finally, a farm diversity score with a minimum attainable score of 0 and a maximum score of 7 was established by summing the scores for the different food crops cultivated and farm animals owned.

### Statistical analysis

Data from the current study were cleaned, coded, and analysed using the Statistical Package for Social Sciences (SPSS, V.21.0, IBM). Descriptive statistics of the sampled population were presented as frequencies and percentages for categorical variables, while means and standard deviation (means ± SDs) were calculated for continuous data. Survey binary logistic regression was employed to assess the factors correlated with the attainment of MDD-W (no = 0/yes = 1). Again, survey binary logistic regression was used to assess the exposure variables correlated with the dietary habits of the girls, that is, the consumption of breakfast, lunch, and supper (≤ 3 days, and >3 days for weekdays and either one of the weekend days, or both weekend days). The girl’s school was included as a stratum in the survey logistic regression analysis. In the analysis, bivariate analysis was first conducted to determine the independent variables correlated with MDD-W and dietary habits. Stepwise backward elimination was specified, and factors with p-value ≤ 0.10 at the bivariate level were included in a multivariable logistic regression model, and a new p-value was set at 0.05. Linear mixed-effect methods were employed to analyse the covariates correlated with the frequency of the consumption of the different food groups in the last month among the girls (mean consumption of cereals, grains, and tubers, pulse, nuts/seeds, animal-source foods, fruits and vegetables, and unhealthy foods). The linear mixed effects analysis included the school as a random intercept, accounting for variation between schools. Multicollinearity between independent factors was examined using tolerance values of < 0.1 and a variance inflation factor of <10 in a linear regression step. A 95% confidence interval with a two-tailed P-value less than 0.05 was deemed statistically significant in all analyses.

Data from the focus groups and in-depth interviews were analysed using an inductive thematic analysis technique. Digital recordings, field notes, and worksheets from the girls were transcribed verbatim. For data quality, transcription of the data was done by two people. The individual transcripts were intensively read and re-examined, and where variations occurred, there was a discussion and an agreement reached. Codes were generated by assigning descriptive labels to relevant text. The codes were categorised, and the different categories were sorted into potential emerging themes. All the key coded responses extracted were then grouped into the various relevant emerging themes, and factors influencing adolescent girls’ food choices were identified. Factors were included based on how frequently, precisely, and comprehensively the quotes pertaining to the factor appeared. All the themes were systematically listed in a theme-count table, and quotations that acted as inferences for the themes found were used to organise the data. One researcher analysed the theme, and this was independently verified by a second researcher.

## Results

### Socio-demographic characteristics of the girls

The descriptive statistics of the 882 adolescent girls from Table 1 show that the mean age of the girls was 12.3 ± 1.9years old. Almost 80% of the girls were pre-menarche. Most (61.2%) of the girls belonged to the Dagomba ethnicity, and about 62.7% were Muslims. Additionally, 92.4% of mothers were non-literate, whereas the mean maternal decision-making index was 5.3 ± 1.4 out of a possible maximum score of 8. The average household size was 12.1 ± 5.1, with a 1.6 ± 1.2 female-to-male sex ratio and 0.5 ± 0.8 household literacy ratio. One-fifth of the girls were from severely food-insecure households, and the mean scores for farm diversity, crop farm diversity, and animal farm diversity were 5.5 ± 1.5, 3.9 ± 1.1 and 1.6 ± 0.6, respectively. Finally, with the quintiles of wealth index, 18.1 % was classified as quintile 1 (poorest) and 19.3% as quintile 5 (richest).

**Table 1.** Demographic and socio-economic characteristics of the adolescent girl (n = 882)

Variables	Mean ± SD/n (%)
Girl-level factors	
Age of girls (years)	12.3 ± 1.9
Ethnicity (n, %)	
Dagomba	540 (61.2)
Konkomba	326 (37.0)
Other	16 (1.8)
Religion n, (%)	
Muslim	553 (62.7)
Christian	316 (35.8)
Africa Traditional	13 (1.5)
Menarche status (n, %)	
Pre-menarche	705 (79.9)
Post-menarche	177 (20.1)
Maternal-related factors	
Maternal literacy (n, %)	
Non-literate	815 (92.4)
Literate	67 (7.6)
Maternal decision index (range 0–8)	5.3 ± 1.4
Household-related factors	
Household size	12.1 ± 5.1
Female-to-male ratio	1.6 ± 1.2
Literacy ratio	0.5 ± 0.8
Household food security status (n, %)	
Food secured	165 (18.7)
Mildly food insecure	237 (26.9)
Moderately food insecure	311 (35.2)
Severely food insecure	169 (19.2)
Quintile of the international wealth index (n, %)	
Quintile 1	160 (18.1)
Quintile 2	187 (21.2)
Quintile 3	193 (21.9)
Quintile 4	172 (19.5)
Quintile 5	170 (19.3)
Household crop farm diversity (range 0–5)	3.9 ± 1.1
Household animal farm diversity (range 0–2)	1.6 ± 0.6
Household Farm Diversity (range 0–7)	5.5 ± 1.5

Unless where specified, values are mean ± SD.

### Dietary diversity and food groups consumed on the previous day by the girls

**Figure 2.**
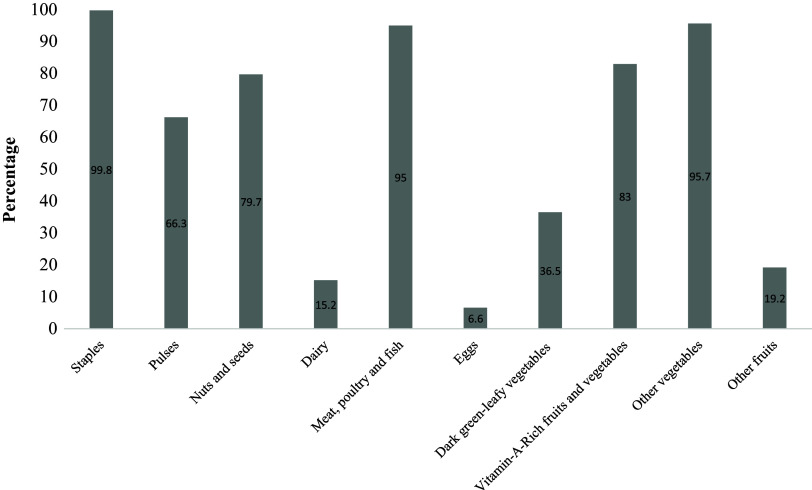
Percentage of girls who consumed each food group in the previous day.

The mean DDS of the girls was 5.9 ± 1.2, with about 90.6% meeting the MDD-W. The most consumed food groups included staples (99.8%), meat, poultry, and fish (95%) and other vegetables (95.7%). The least consumed food groups were dairy products (15.2%) and eggs (6.6%) (Fig. 2).

### Eating habits of the adolescent girls

As depicted in Table 2, 90.6% of the girls had breakfast on weekends (Saturday and Sunday), whereas 95.6% had breakfast for more than 3 days during weekdays (Monday-Friday). Again, during the weekend, 92% ate lunch on both weekend days, and 96.5% had lunch more than 3 days during weekdays. Dinner was consistently consumed by almost all the girls on both weekend days and at least thrice on weekdays. Generally, nearly all the girls reported consuming all three main meals: breakfast, lunch, and dinner on weekdays. A similar pattern was observed over the weekends.

**Table 2.** Eating habits of the adolescent girls

Variables	n (%)
Breakfast (weekend)	
Either one of the weekend days	83 (9.4)
Both days	799 (90.6)
Breakfast (weekdays)	
≤3 days	39 (4.4)
>3 days	843 (95.6)
Lunch (weekend)	
Either one of the weekend days	71 (8.0)
Both days	811 (92.0)
Lunch (weekdays)	
≤3 days	31 (3.5)
>3 days	851 (96.5)
Supper (weekend)	
Either one of the weekend days	56 (6.3)
Both days	826 (93.7)
Supper (weekdays)	
≤3 days	9 (1.0)
>3 days	873 (99.0)
The girl ate all 3 meals on weekdays	
Yes	874 (99.1)
No	8 (0.9)
The girl ate all 3 meals during the weekend	
Yes	869 (98.5)
No	13 (1.5)

### Frequency of consumption of food groups in the last month by the girls

Over the past 30 days, staples such as cereals, grains, tubers, and plantains were consumed daily, while pulses (beans, peas, lentils) and nuts/seeds were consumed 15.2 ± 7.4 days (Table 3). Additionally, the mean number of days the girls consumed ASFs, F&V, and unhealthy foods were 8.6 ± 3.9, 11.7 ± 6.0, and 6.9 ± 4.4 days, respectively. Compared to vegetables, fruit were the least consumed foods.

**Table 3.** Frequency of the consumption of food groups in the last month by the girls

Variables	Number of days in the last month (Mean ± SD)
Staples (cereals, grains, and tubers)	29.4 ± 3.5
Pulses (beans, peas, lentils) and nuts/seeds	15.2 ± 7.4
Animal-source foods (dairy, eggs, meat, and fish)	8.6 ± 3.9
Fruits and vegetables	11.7 ± 6.0
Vegetables	17.1 ± 10.4
Fruits	6.2 ± 5.8
Unhealthy foods (sweets, SSBs, savoury and fried snacks)	6.9 ± 4.4

#### Foods commonly eaten at mealtimes

The in-depth interviews revealed common breakfast choices, such as porridge, tea with bread, tuo-zaafi (TZ) (a stiff porridge made from maize flour), and rice dishes, as outlined in Table 4. TZ was a daily choice for lunch (100%) and dinner (93.3%). Rice dishes (50-60 %) and yam or fufu (pounded yam, 16.6%) were also commonly eaten. Pigeon peas were mainly consumed during lunch (16.7%), with some also reporting it for dinner (10.0%). Interestingly, biscuits/pastries (63.3%) and sweets (56.7%) ranked as the top two snack options, with fruits (50.0%) coming in third. Ice cream/yoghurt (36.7%) and sugar-sweetened beverages (26.7) were also popular snack choices. The focus group discussions confirmed the patterns observed in the in-depth interviews regarding the foods commonly consumed at mealtimes.

**Table 4.** Foods commonly eaten at mealtimes; qualitative in-depth interviews (n = 30)

	Theme Frequency	Percentage (%)
Breakfast		
Porridge	26	86.7
Tea with bread	16	53.3
‘Tuo Zaafi’	15	50
Rice dishes	9	30
Others	8	26.7
**Lunch**		
‘Tuo Zaafi’	30	100
Rice dishes	18	60
Pigeon pea	5	16.7
Yam	4	13.3
Corn meal (‘yoroyoro’)	3	10
Others	7	23.3
**Supper**		
‘Tuo Zaafi’	28	93.3
Rice dishes	15	50
‘Fufu’	5	16.6
Yam	4	13.3
Banku	3	10
Pigeon pea	3	10
Others	3	10
**Snack**		
Biscuits /Pastries	19	63.3
Sweets	23	76.7
Fruits	15	50
Ice cream/Yoghurt	11	36.7
Soft drinks	8	26.7
Local beverages	8	26.7
Others	4	13.3

### Factors associated with the dietary diversity (low/high) among the girls

In the bivariate analysis (Table S1), independent variables such as ethnicity (*p* = 0.014), religion (*p* = 0.002), menarche status (*p* = 0.070), household food security status (*p* = 0.005), and household size (*p* = 0.007) were identified as the significant factors associated with achieving the MDD-W. However, the multivariable logistic regression analysis (Table 5) indicated that household size and food security were significantly associated with achieving the MDD-W. Specifically, girls from households with moderate to severe food insecurity were 68% less likely to achieve MDD-W (Adjusted Odds Ratio (AOR) = 0.32; 95% CI: 0.14, 0.72). Similarly, girls from mildly food-insecure households had a 58% reduced likelihood of meeting the MDD-W (AOR = 0.42; 95% CI: 0.17, 1.01). Unexpectedly, a larger household size was positively associated with achieving the MDD-W, showing an 8% increase in odds with each additional household member (AOR = 1.08; 95% CI: 1.02, 1.13).

**Table 5.** Factors associated with Minimum Dietary Diversity (MDD-W > 5) among the adolescent girls: multivariate logistic regression analysis

Variables	OR (95 % C.I)	P-value
Household size	1.08 (1.02, 1.13)	0.005
Household food security status		
Food secured	Ref	
Mildly food insecure	0.42 (0.17, 1.01)	0.054
Moderately/ Severely food insecure	0.32 (0.14, 0.72)	0.006
Model fit statistics		
Nagelkerke R Square	0.043	
-2 Log-likelihood ratio	532.598	
Wald test	24.962	0.000

### Factors associated with the eating habits during weekdays and weekends among the girls

In the bivariate analysis, significant predictors of breakfast consumption more than 3 days on weekdays included the girl’s age, religion, and household food security. Mild and moderate/severe food insecurity was also positively correlated with higher odds of consuming lunch at least 3 days on weekdays (Table S2). The household wealth index was associated with consuming supper more than 3 days on weekdays. However, except for the girl’s age, which was negatively associated with reduced odds of eating breakfast more than 3 days during weekdays, no other factors were identified for breakfast, and none were identified for lunch and dinner during weekdays at the multivariate level (Table 6). Likewise, only crop diversity (Table S3) correlated with increased odds of eating supper on both weekend days and no significant factors were identified for breakfast and lunch during the weekend (Table 6).

**Table 6.** Factors associated with eating habits among the adolescent girls: multivariate logistic regression analysis

Variables	Weekdays^a^		Weekends^a^	
	Breakfast^b^		Supper^b^	
	OR (95 % C.I)	P-value	OR (95 % C.I)	P-value
Age of girls (years)	0.77 (0.65, 0.90)	0.001		
Crop diversity			1.63 (1.34, 1.97)	0.000
Model fit statistics				
Nagelkerke R Square	0.08		0.07	
−2 Log-likelihood ratio	297.57		395.41	
Wald test	10.54		24.94	

a The odds of eating the meal more than 3 days during the weekday and eating the meal on both weekend days.

b Apart from breakfast on weekdays and dinner on weekends, our analysis did not identify any significant covariates at the multivariate level for lunch and dinner during weekdays and breakfast and lunch during the weekend.

### Factors associated with the frequency of consumption of some food groups among the girls

At the bivariate level, maternal decision-making index was significantly correlated with F&V intake (Table S4). Ethnicity, farm diversity, animal farm diversity, food security, and wealth index were factors correlated with ASF intake. Muslim religion, maternal non-literacy, household size, food security, wealth index, farm diversity, and crop and animal diversity significantly correlated with the consumption of unhealthy items. Also, the wealth index, farm diversity, crop diversity, and animal diversity were significantly associated with pulses/nuts and seeds consumption. Mildly food insecure, maternal decision-making index, farm diversity, crop diversity, animal diversity, and wealth index were also predictors of the consumption of cereals, grains, and tubers (Table S4). While food insecurity was inversely associated with eating fruits, it was positively associated with eating more vegetables in the last 30 days (Table S5).

In the backward multivariate regression, moderate/severe food insecurity and Konkomba ethnicity were negatively and significantly correlated with the intake of ASFs. Also, maternal non-literacy and household food security were factors negatively correlated with the consumption of unhealthy foods. Quintiles 3 and 5 of the household wealth indices were positively associated with the consumption of staples. The multivariate results also showed that mild and moderate/severe food insecurity was inversely associated with the intake of fruits but positively associated with eating more vegetables in the last month. Finally, no significant multivariate predictors were found for pulses/nuts and seeds intake besides combined F&V (Table 7).

**Table 7.** Factors influencing frequency of food group consumption among the adolescent girls: multivariate linear mixed-effect model analysis

Variables	Animal source foods		Unhealthy foods		Cereals, grains, and Tubers		Fruits		Vegetables	
	Estimate (95 % C.I)	P-value	Estimate (95 % C.I)	P-value	Estimate (95 % C.I)	P-value	Estimate (95 % C.I)	P-value	Estimate (95 % C.I)	P-value
Maternal literacy										
Literate			Ref							
Non-literate			−1.86 (-2.94, -0.78)	0.001						
Household food security status										
Food secured	Ref		Ref				Ref		Ref	
Mildly food insecure	−0.45 (-1.21,0.30)	0.239	−1.07 (-1.94, -0.21)	0.015			−2.02 (-3.15, -0.89)	0.0001	2.93 (0.86, 4.99)	0.005
Moderately/ Severely food insecure	−1.59 (-2.27-0.92)	0.000	−1.88 (-2.63, -1.10)	0.000			−2.40 (-3.41, -1.39)	0.0001	3.20 (1.33, 4.99)	0.001
Ethnicity										
Other	Ref									
Dagomba	−1.61 (-3.49,0.28)	0.95								
Konkomba	−2.02 (-3.93, -0.12)	0.037								
Quintiles of wealth index										
Quintile 1					Ref					
Quintile 2					0.56 (-0.18,0.21)	0.135				
Quintile 3					0.99 (0.26, 1.73)	0.008				
Quintile 4					0.71 (-0.05,1.46)	0.068				
Quintile 5					1.13 (0.37, 1.88)	0.004				
Information criteria										
-2 Restricted Log Likelihood	4848.84		5086.65		4712.07		5569.21		6619.75	
AIC	4850.84		5088.65		4714.07		5571.21		6621.75	

Fruits and Vegetables, and Pulses and Nuts/Seeds were excluded from the table due to the lack of statistically significant factors influencing their consumption.

#### Reasons why adolescent girls eat what they eat

The in-depth interviews identified four main reasons influencing the dietary choices of the adolescent girls: health reasons (46.7%), availability (23.3%), cravings (16.7%), and taste (13.3%). The focus group discussions further confirmed that health and food availability were the primary factors determining their food choices. Below are the reasons provided by two girls during the in-depth interviews for why they eat the foods they eat:

*Some of them give me energy, and some of them are sweet. For instance, you get energy when you feel weak and drink the run energy drink. Eating eggs is healthy because they can help increase your blood levels when you lack enough.*

Source: *[(Pupil) – Zakpalsi]*

*My mother and others at home always cook it*

Source*: [(Pupil) – Kpabia]*

### Food likes and dislikes of adolescent girls and reasons for likes and dislikes

The in-depth interviews revealed that sweets and F&V were each favoured by 13.3% of the girls, followed by TZ (10.0%), fufu (6.7%), and rice (6.7%). However, the focus group discussions indicated a preference shift, with local beverages replacing TZ as the third most liked food. Interestingly, many girls disliked TZ (26.7%) and fruits/vegetables (20%), despite some favouring them. Other commonly disliked foods included beans/pigeon peas and corn meal (yoroyoro). The focus groups specifically identified TZ with dried baobab leaf soup (‘kuka’) as the food the participants disliked the most.

The primary reasons adolescent girls favoured certain foods were due to food tolerance (36.7%), perceived health benefits (20.0%), and taste (6.7%). Conversely, food aversions also related to food intolerance (23.3%), fatigue from repetitive consumption of the same food (20.0%), disliking the taste (13.3%), texture/appearance (10.0%), or due to a lack of appetite (3.3%). Focus group discussions further highlighted taste, satiety, and food intolerance as primary factors influencing food preferences, while repetitive consumption and texture were commonly cited reasons for disliking foods. The statements below outline the various reasons behind the girls’ food preferences and aversions.

*I don’t like foods that taste bitter or too sweet. Yellow melon, “kontomire” (cocoyam leaves), and bitter leaves- I don’t like them because they are bitter. But the yellow melon is too sweet, so I don’t like it.*

Source: *[(Pupil) – Sanzee]*

*Beans, I am allergic to it. My stomach pains when I eat it.*

Source: [(Pupil) – Kulinkpegu]

*I am tired of eating T.Z. because we eat it daily.*

Source: *[(Pupil) – Jimle]*

*Sometimes, when I eat T.Z., I feel nauseous, and I vomit; I am allergic to it.*

Source: *[(Pupil) – Kulinkpegu]*

## Discussion

The purpose of this study was to assess the dietary patterns, dietary diversity, eating habits, and the factors influencing these, as well as to explore the reasons behind the food choices of adolescent girls in the Mion district of Northern Ghana. The average DDS of the girls was 5.9 ± 1.2, which was a bit higher than the 3.8 ± 0.8 found by Wiafe *et al.* ^(28)^ in the Ashanti Region of Ghana and the 4.9 ± 1.47 that was found in a previous study in Addis Ababa, Ethiopia.^(29)^ Surprisingly, most of the girls in the present study were found to have achieved the MDD-W compared to the study of Wiafe *et al*.^(28)^ and the study of Worku *et al.*.^(29)^ The observed variations in dietary intake may stem from cultural and socio-demographic differences across study settings. Notably, in comparison to Wiafe *et al*.,^(28)^ our sample demonstrated higher consumption of pulses (66.3% vs 10.2%), nuts and seeds (77.7% vs 43.1%), and other vitamin A-rich fruits and vegetables (83.0% vs 2.2%). The timing of data collection could also explain these differences. It should be noted that although our study coincided with the beginning of the crop harvest period, moderate-to-severe household food insecurity was still prevalent. Since food insecurity was not evaluated in the study by Wiafe and colleagues,^(28)^ direct comparisons cannot be made.

In conformity with previous studies^(30,31)^ food insecurity was found to decrease the odds of achieving the MDD-W and was associated with decreased consumption of unhealthy foods, which contrasted with a Brazilian study.^(31)^This could be due to the fact that most rural communities’ diets are predominantly starchy staples (cereals, grains, and tubers), as they cultivate these crops on subsistent basis to feed the family with little or no variety. Moreso, households that are severely food insecure are likely to be poor and may not have enough purchasing power to consume unhealthy foods. The inverse relationship between food insecurity and the MDD-W was reinforced by findings from in-depth interviews and focus group discussions, which highlighted food availability as a key factor influencing dietary choices. Food availability ranked as the second most important determinant of the girls’ food choices, with their diets largely dependent on what was readily accessible and provided to them.

Additionally, food insecurity correlated with reduced intake of ASFs, consistent with findings from Ethiopia^(32)^ and was inversely associated with fruit intake but correlated with increased vegetable intake. Notably, higher household wealth was linked to increased consumption of staple foods, likely due to prioritising fulfilling basic caloric needs. Moreso, ASFs^(33)^ and fruits^(34)^ are known to be more expensive in Ghana, which explains why ASFs are less likely to be purchased for household consumption, with vegetables preferred over fruits. Cultural dietary patterns may further explain the differential influences of food insecurity on fruit and vegetable intake, with fruits often consumed as snacks and desserts. In contrast, alongside staple foods, vegetables are common in soups and sauces, making them more integrated into daily diets in Ghana.

Interestingly, the in-depth interviews revealed that one-fifth of the adolescent girls disliked F&Vs, adding context to the quantitative results. This aversion stemmed from several factors: taste preferences, exposure, and preparation methods. Consistent with our findings, studies in India and Nigeria^(35,36)^ also showed that several personal-level factors, including taste, preparation and exposure, influenced food choice among adolescent girls. Such dislikes could compound the challenges posed by economic constraints, further limiting F&V consumption even when they are available. Understanding the connection between taste, eating habits, and food consumption is important during this critical phase of growth and development. Therefore, promoting healthy eating that considers taste preferences is essential for improving the dietary habits of adolescent girls.^(37)^ Addressing these preferences through targeted nutrition education, improved food preparation methods, and strategies to make F&Vs more appealing could help mitigate these barriers and improve overall dietary quality.

Although health considerations were cited as the most important factor, the lack of available healthy options may constrain the girls’ choices, overriding health priorities. Research consistently highlights the influence of food availability on adolescent eating behaviour. For example, studies in the United States have shown that access to nutritious, home-prepared meals correlates with increased fruit and vegetable consumption and reduced intake of soda and snack foods.^(37)^ Similarly, food availability significantly impacts dietary habits among adolescent girls in Nigeria^(38)^ and the Benin Republic.^(39)^ These findings underscore the critical need for strategies that enhance access to affordable, nutrient-rich foods to support healthier dietary patterns among adolescents.

Contrary to the literature, which shows that a large family is a barrier affecting diet quality and food security,^(40–42)^ household size was positively associated with achieving the MDD-W. A larger family size results in higher demands on the household’s adults, increasing food expenses and decreasing the quantity, quality, and variety of foods consumed. In the present study, most of the girls belonged to a household with a larger family size. However, families with most of their members employed could also be less poor, as a broader workforce can participate in more agricultural practices, boosting output or earnings from paid labour, thus enhancing their purchasing power and a higher DDS; this may explain the finding.

The findings of multiple studies indicate that children and adolescents who regularly eat a healthy breakfast have better cognitive health and nutritional status and lower plasma cholesterol levels.^(38,43)^ Interestingly, older age was associated with lower odds of consuming breakfast over 3 days during the weekdays in the present study. In line with the results of this study, Onyiriuka *et al*.^(38)^ found that breakfast was the most frequently skipped meal, with 30% of girls under 14 years, 50% aged 14–16 years, and 59% older than 16 years skipping meals. This could be because some adolescents no longer receive the same level of attention and care as children, which could explain their autonomous motivation and sense of control over their eating habits.

Again, there was a correlation between crop diversification and a higher likelihood of having dinner at the weekend in the present study. Generally, supper or the evening meal is one of the most important and nutritious meals.^(43)^ In Northern Ghanaian culture, supper serves as a key household meal, typically prepared at home. Adolescents’ dietary choices at home in the evening are influenced by various circumstances, primarily influenced by what adults provide and expect them to consume.^(44)^ The positive association between crop diversity and the consumption of supper during the weekend is consistent with the general view that farm households’ daily intake reflects the variety of crops they cultivate.^(45)^ Currently, there is no research on the relationship between crop diversity and adolescent girls’ food habits, and it is unclear what mechanism underlies the association. However, the predominant crops cultivated in Mion are cereals and root tubers; these are often consumed as staples daily, as found in the present study. Research shows that what is cultivated is expected to reflect in one’s diet.^(46)^ It was, however, surprising that the same association was not found on weekdays.

In the current study, belonging to the Konkomba ethnic group was negatively associated with consuming ASFs. Ethnicity plays a major role in the food choices and habits of populations. For instance, among the Dagaare people in Northern Ghana, pregnant women are not allowed to eat baobab leaves, and offering protein products to their children, such as eggs and meat, raises concerns that it could encourage theft.^(42)^ Contrary to the current study, Drewnowski and colleagues^(47)^ discovered that ethnicity was related to the consumption of animal protein, with Indians consuming more dairy products and eggs, Malaysians consuming more seafood, and Chinese people consuming more meat. In addition to cost, in rural areas within the study region, ASFs are typically reserved for special family or public occasions. They are viewed as enjoyable treats rather than essential components of the daily diet. This cultural perception likely contributes to the observed inverse association between ASF consumption and the Konkomba ethnic group, reflecting socio-cultural norms that prioritise ASFs for celebratory contexts rather than routine dietary practices.

Maternal non-literacy was negatively correlated with the intake of unhealthy items. In contrast with the present study’s findings, several studies discovered that girls whose mothers were not well-educated were more likely to consume high-fat, energy-dense drinks, added sugar, and salty foods.^(48,49)^ Evidence indicates that children with healthy lifestyles and appropriate dietary choices are more likely to have mothers with high educational levels. A high maternal educational level probably predicts better nutritional awareness, food choices, and parenting behaviours.^(50)^ However, the association between maternal non-literacy and the lower consumption of unhealthy foods could be explained by the study’s qualitative findings, which showed that most girls eat what is cooked at home with little out-of-home food consumption.

The study was conducted in a rural area where agriculture is the primary livelihood, with a low average household wealth index. Despite this, girls from households in the middle and upper wealth quintiles were more likely to consume staples, indicating limited availability and access to even basic staple foods. This finding contrasts with a study by Agdeppa *et al.* ^(51)^ noted that adolescents from poorer socio-economic backgrounds in the Philippines consumed more rice, pasta, and other grain products than those from wealthier backgrounds.

Adolescents are more likely to engage in healthy eating behaviours if they perceive immediate health benefits, such as improved skin, stronger muscles, and enhanced gut health.^(52)^ The present study showed that dietary choices among adolescent girls are often driven by their perceived health perceptions, which was similar to a study that found that Polish girls who are more health-conscious tend to adopt healthier diets.^(49)^ Similarly, Sedibe *et al*.^(50)^ found that adolescent girl’s dietary choices are shaped by their understanding of what constitutes a healthy diet. This suggests that health considerations are crucial in shaping teenage girl’s dietary habits, ultimately impacting their long-term health and well-being.

Additionally, the present study found that cravings can significantly influence food habits among adolescent girls. A study found that the relationship between food cravings and body image is closely tied to healthy eating habits in adolescent girls.^(53)^ Moreover, food addiction can exacerbate insane food cravings, making healthy eating a challenge for teens, especially for those who did not experience a food addiction before weight gain.^(54)^ Similarly, increased trait food cravings have been linked to the consumption of harmful foods among adolescent girls.^(55)^ In the present study, the adolescent girls also indicated being ‘fed-up’ as a reason for food dislikes because certain foods, such as T.Z., a staple recipe, were served at home almost daily.

Quantitative data showing limited F&V intake aligns with qualitative insights, suggesting that economic and personal preferences shape dietary patterns. The dislike of fruits, a key theme from the qualitative data results, may partly explain why F&V consumption, especially fruit, was low among girls. The Global School-based Student Health Survey also indicates that most adolescents worldwide consume fewer F&V than the recommended amounts.^(56)^ It is suggested that healthy family meals are crucial in increasing F&V intake among teenagers and forming their dietary habits.^(57)^ The cultural dietary patterns and affordability mentioned earlier may also explain why fruit intake and preference for fruits were lower than vegetable intake and preference in our study.

### Strengths and limitations

The main strength of this study lies in its mixed-methods approach, combining quantitative and qualitative data for triangulation. Additionally, the study benefits from a larger sample size relative to most adolescents’ dietary intake research and incorporates a range of explanatory variables across different levels. However, the study also has some limitations. There is a possibility that some data, such as meal habits, dietary diversity, and household food insecurity, may be subject to short-term memory bias, potentially leading to underestimation or overestimation of the results. To mitigate this, mothers assisted adolescent girls in recalling household food insecurity and food intake when necessary. However, mothers were not included in the qualitative interviews, which may be a limitation as we may have missed the mothers’ perspectives on drivers of the girls’ food choices. Contrariwise, excluding mothers in these interviews ensured that the adolescent girls expressed their perspectives independently, allowing for a more authentic and unfiltered understanding of their experiences and perceptions related to food choice and dietary habits. This approach likely provided deeper insight into the girls’ own decision-making processes, challenges, and coping strategies without external influence. Because of the cross-sectional survey design, associations were estimated, and causality could not be inferred from the results. Lastly, only females from one district were sampled; therefore, the findings of this study might not be generalisable to males or Ghana as a whole. Although the sample was school-going adolescent girls, nearly all Ghanaian children are now enrolled in school. Therefore, the study population may represent all rural teenage girls in the Mion district and similar settings in Ghana.

### Conclusion

The study indicated that most of the girls achieved the MDD-W. It also showed that nearly all participants consumed three meals daily throughout the week. Staples were a daily part of their diet, while fruits and unhealthy foods were consumed less frequently. Household food security emerged as a significant predictor of various dietary outcomes among adolescent girls, including low MDD-W and reduced consumption of ASF, fruits, and unhealthy foods. Still, food security was associated with increased vegetable consumption. Our study also underscores the multifaceted influences on dietary diversity, eating habits, and consumption patterns among adolescent girls, encompassing age, ethnicity, household SES, household size, maternal literacy, and crop diversity. Insights from focus group discussions and in-depth interviews shed light on the varied reasons behind adolescent girls’ food choices, including health, taste, cravings, availability, and hunger satisfaction. Overall, the study highlights that adolescent girls’ dietary diversity and eating habits are significantly shaped by food security, socio-economic, household, and cultural factors, as well as personal preferences and food availability. We therefore recommend the implementation and improvement of school-based nutrition initiatives such as the ongoing school feeding programme. Encouraging the sale of healthier food options like fruits and vegetables and discouraging the sale of unhealthy foods in school canteens, as well as educational campaigns could help enhance adolescent girls’ overall nutrition and health. Also, food security, livelihood improvement and empowerment interventions in the form of capacity building both physically and economically and support for diversified agriculture could enhance household’s access to fruits and animal-source foods.

## Supporting information

Al-Hassan et al. supplementary material Al-Hassan et al. supplementary material

## Supplementary material

The supplementary material for this article can be found at https://doi.org/10.1017/jns.2025.17
