# Supplementary material for: Dietary diversity, eating habits, dietary patterns, food choice, and associated factors among adolescent girls: a convergent parallel mixed-method study in the Mion District of Ghana
Source: J Nutr Sci. 2025 Mar 26;14:e28. doi: 10.1017/jns.2025.17 (PMC11955308; doi:10.1017/jns.2025.17)
Supplement: Al-Hassan et al. supplementary material [file S2048679025000175sup001.pdf]

Table S1: Factors Associated with Dietary Diversity (Low/High) among the Adolescent Girls: Bivariate Logistic Regression Analysis

Variables	OR (95 % C.I)	P-value
Age of girls (years)	0.91 (0.81, 1.02)	0.109
**Ethnicity**		0.014
Dagomba	Ref	
Konkomba	0.52 (0.32, 0.81)	0.004
Other	1.17 (0.15, 9.07)	0.882
**Religion**		0.002
Muslim	Ref	
Christian	0.50 (0.31, 0.78)	0.003
Other	0.42 (0.09, 1.94)	0.266
**Menarche Status**		
Pre-menarche	Ref	
Post-menarche	0.62 (0.37, 1.04)	0.070
**Maternal literacy**		0.571
Non-literate	Ref	
Literate	0.76 (0.30, 1.95)	0.571
Maternal decision index	0.98 (0.83, 1.16)	0.828
Female-to-male (Sex) ratio	0.93 (0.78, 1.12)	0.409
Household size	1.07 (1.02, 1.13)	0.007
Literacy ratio	1.04 (0.78, 1.38)	0.799
Farm diversity	1.01 (0.87, 1.17)	0.909
Crop diversity	0.98 (0.79, 1.21)	0.846
Animal Diversity	1.22 (0.88, 1.70)	0.231
**Household food security status**		0.005
Food secured	Ref	
Mildly food insecure	0.46 (0.19, 1.10)	0.080
Moderately food insecure	0.37 (0.16, 0.86)	0.021
Severely food insecure	0.30 (0.12, 0.71)	0.007
**Quintile of the international wealth index**		0.227
Quintile 1	Ref	
Quintile 2	0.66 (0.32, 1.36)	0.264
Quintile 3	0.62 (0.31, 1.27)	0.191
Quintile 4	0.10 (0.45, 2.19)	0.996
Quintile 5	1.42 (0.60, 3.33)	0.426

Table S2: Factors Associated with Dietary Habits during Weekdays among the Adolescent Girls: Bivariate Logistic Regression Analysis

Variables	Breakfast		Lunch		Supper	
	**OR (95 % C.I)**	**P-value**	**OR (95 % C.I)**	**P-value**	**OR (95 % C.I)**	**P-value**
Age of girls (years)	0.77 (0.66, 0.91)	0.001	1.01 (0.83, 1.23)	0.904	0.88 (0.64, 1.23)	0.463
**Ethnicity**						
Dagomba	Ref.	0.206	Ref.	0.390	Ref.	1.000
Konkomba	0.56 (0.29, 1.06)	0.076	1.69 (0.74, 3.84)	0.211	Unreliable	
Other	Unreliable		0.64 (0.08, 5.04)	0.669	Unreliable	
**Religion**						
Muslim	Ref.	0.063	Ref.	0.248	Ref.	1.000
Christian	0.59 (0.30, 1.14)	0.116	1.92 (0.81, 4.52)	0.137	Unreliable	
Other	0.20 (0.04, 0.95)	0.042	0.52 (0.07, 4.18)	0.539	Unreliable	
**Menarche Status**						
Pre-menarche	Ref.		Ref.		Ref.	
Post-menarche	0.72 (0.34, 1.49)	0.376	1.05 (0.42, 2.59)	0.920	2.02 (0.25, 16.26)	0.509
**Maternal literacy**						
Non-literate	Ref.		Ref.		Ref.	
Literate	1.85 (0.70, 4.91)	0.215	0.54 (0.18, 1.59)	0.263	0.65 (0.08, 5.31)	0.691
Maternal decision index	0.98 (0.78, 1.24)	0.856	1.00 (0.78, 1.30)	0.989	1.16 (0.74, 1.81)	0.525
Female-to-male (Sex) ratio	0.89 (0.71, 1.09)	0.268	0.98 (0.74, 1.31)	0.900	1.01 (0.59, 1.73)	0.985
Household size	1.01 (0.95, 1.08)	0.781	0.99 (0.93, 1.07)	0.947	0.94 (0.84, 1.05)	0.286
Literacy ratio	1.09 (0.70, 1.71)	0.699	1.14 (0.67, 1.95)	0.625	1.05 (0.44, 2.50)	0.905
Farm diversity	1.12 (0.92, 1.35)	0.254	1.09 (0.88, 1.35)	0.446	0.74 (0.40, 1.37)	0.337
Crop diversity	1.11 (0.84, 1.46)	0.466	1.15 (0.85, 1.55)	0.372	0.61 (0.25, 1.47)	0.265
Animal Diversity	1.50 (0.97, 2.32)	0.066	1.19 (0.71, 2.01)	0.513	0.89 (0.30, 2.63)	0.835
**Household food security status**		0.000		0.583		0.723
Food secured	Ref.		Ref.		Ref.	
Mildly food insecure	0.15 (0.02, 1.23)	0.078	2.48 (0.89, 6.97)	0.084	0.72 (0.06, 7.97)	0.786
Moderately food insecure	0.14 (0.02, 1.08)	0.059	3.28 (1.17, 9.19)	0.024	0.37 (0.04, 3.22)	0.370
Severely food insecure	0.06 (0.01, 0.45)	0.006	1.15 (0.45, 2.90)	0.772	1.02 (0.06, 16.52)	0.986
**International wealth index**	0.99 (0.96, 1.02)	0.550	1.01 (0.98, 1.04)	0.620	0.95 (0.89, 1.00)	0.056

Table S3: Factors Associated with Dietary Habits during Weekend Days among the Adolescent Girls: Bivariate Logistic Regression Analysis

Variables	Breakfast		Lunch		Supper	
	OR (95 % C.I)	P-value	OR (95 % C.I)	P-value	OR (95 % C.I)	P-value
Age of girls (years)	0.99 (0.88, 1.12)	0.914	0.99 (0.88, 1.14)	0.992	0.01 (0.87, 1.17)	0.890
**Ethnicity**		0.144		0.012		0.001
Dagomba	Ref.		Ref.		Ref.	
Konkomba	1.42(0.87, 2.32)	0.161	2.53 (1.38, 4.62)	0.003	3.79 (1.77, 8.13)	0.001
Other	1.77 (0.23, 13.65)	0.584	0.79 (0.18, 3.59)	0.764	1.43 (0.19,11.07)	0.732
**Religion**		0.169		0.003		0.001
Muslim	Ref.		Ref.		Ref.	
Christian	1.43 (0.87, 2.34)	0.162	2.68 (1.44, 4.98)	0.002	4.20 (1.88, 9.39)	0.000
Other	1.41 (0.18, 11.01)	0.746	1.38 (0.18, 10.80)	0.760	1.14 (0.15, 8.96)	0.900
**Menarche Status**						
Pre-menarche -	Ref.		Ref.		Ref.	
Post-menarche	0.90 (0.52, 1.56)	0.699	1.26 (0.66, 2.39)	0.488	1.33 (0.64, 2.78)	0.442
**Maternal literacy**		0.894		0.455		0.158
Non-literate	Ref.		Ref.		Ref.	
Literate	1.06 (0.44, 2.53)	0.894	0.73 (0.32, 1.66)	0.455	0.55 (0.24, 1.26)	0.158
Maternal decision index	0.85 (0.72, 1.01)	0.061	0.87 (0.72, 1.04)	0.128	0.86 (0.70, 1.05)	0.149
Female-to-male (Sex) ratio	0.93 (0.78, 1.09)	0.358	0.90 (0.76, 1.06)	0.209	0.87 (0.73, 1.04)	0.121
Household size	1.00 (0.96, 1.05)	0.879	0.99 (0.95, 1.04)	0.845	1.00 (0.95, 1.05)	0.938
Literacy ratio	0.87 (0.70, 1.09)	0.230	0.86 (0.68, 1.08)	0.187	0.87 (0.68, 1.13)	0.301
Farm diversity	1.31 (1.16, 1.48)	0.000	1.25 (1.10, 1.43)	0.001	1.35 (1.18, 1.55)	0.000
Crop diversity	1.39 (1.17, 1.66)	0.000	1.43 (1.19, 1.72)	0.000	1.63 (1.34, 1.97)	0.000
Animal Diversity	1.79 (1.33, 2.42)	0.000	1.40 (0.99, 1.97)	0.051	1.38 (0.95, 2.01)	0.093
**Household food security status**		0.531		0.540		0.016
Food secured	Ref.		Ref.		Ref.	
Mildly food insecure	0.80 (0.39, 1.62)	0.529	1.23 (0.61, 2.47)	0.557	1.01 (0.49, 2.05)	0.987
Moderately food insecure	0.80 (0.41, 1.58)	0.523	1.41 (0.72, 2.77)	0.317	1.60 (0.77, 3.34)	0.207

Severely food insecure	0.77 (0.36, 1.63)	0.487	1.19 (0.56, 2.52)	0.652	3.04 (1.07, 8.65)	0.037
**Quintile of the international wealth index**		0.902		0.971		0.866
Quintile 1	Ref.		Ref.		Ref.	
Quintile 2	0.72 (0.36, 1.46)	0.360	1.19 (0.55, 2.57)	0.667	1.18 (0.50, 2.80)	0.706
Quintile 3	1.14 (0.53, 2.43)	0.739	1.14 (0.53, 2.43)	0.739	1.22 (0.52, 2.90)	0.650
Quintile 4	1.00 (0.47, 2.15)	0.993	1.08 (0.50, 2.35)	0.842	1.08 (0.46, 2.57)	0.861
Quintile 5	0.86 (0.41, 1.81)	0.697	1.07 (0.49, 2.32)	0.867	0.97 (0.42, 2.27)	0.948

Table S4: Factors Associated with Frequency of Consumption of Food Groups among the Adolescent Girls: Bivariate Linear Mixed-Effect Model Analysis

	**Fruits and Vegetables**		**Animal source foods**		**Unhealthy foods**		**Pulses and Nuts/Seeds**		**Cereals, grains, and tubers**	
**Variables**	**Estimate (95 % C.I)**	**P-value**	**Estimate (95 % C.I)**	**P-value**	**Estimate (95 % C.I)**	**P-value**	**Estimate (95 % C.I)**	**P-value**	**Estimate (95 % C.I)**	**P-value**
Age of girls (years)	0.08 (-0.13,0.29)	0.461	0.09 (-0.04,0.23)	0.178	0.06 (-0.09,0.22)	0.417	-0.17(-0.44,0.09)	0.191	0.07(-0.05, 0.19)	0.273
**Ethnicity**										
Dagomba	-0.62 (-3.61,2.36)	0.683	-1.69 (-3.60,0.22)	0.083	0.64 (-1.50, 2.78)	0.557	2.49(-1.19, 6.16)	0.184	-0.48(-2.23,1.27)	0.592
Konkomba	-0.57 (-3.59,2.44)	0.709	-2.29 (-4.22,0.36)	0.020	-1.53 (-3.69,0.63)	0.165	1.58(-2.13, 5.29)	0.402	-0.86(-2.63,0.91)	0.340
Other	Ref		Ref		Ref		Ref		Ref	
**Religion**										
Muslim	-2.61 (-5.91,0.69)	0.121	0.46 (-1.65, 2.58)	0.667	2.03 (-0.34,4.40)	0.093	0.52(-3.55, 4.59)	0.801	-0.47(-2.40,1.47)	0.636
Christian	-2.72 (-6.05,0.60)	0.109	-0.15(-2.29,1.99)	0.890	-0.09(-2.48,2.29)	0.939	-0.26(-4.37,3.84)	0.900	-0.89(-2.84,1.07)	0.373
Other	Ref		Ref		Ref		Ref		Ref	
**Menarche Status**										
Pre-menarche	-0.18(-1.17, 0.81)	0.724	-0.35(-0.98,0.29)	0.287	0.01(-0.72, 0.74)	0.977	0.16(-1.05, 1.39)	0.791	0.07(-0.51, 0.65)	0.811
Post-menarche	Ref		Ref		Ref		Ref		Ref	
**Maternal literacy**										
Non-literate	-0.39(-1.88, 1.11)	0.611	-0.31(-1.27,0.65)	0.528	-1.95(-3.04, -0.85)	0.000	0.01(-1.83, 1.86)	0.991	-0.43(-1.31,0.44)	0.332
Literate	Ref		Ref		Ref		Ref		Ref	

Maternal decision index	-0.25(-0.53, 0.04)	0.087	-0.15(-0.33,0.03)	0.104	0.12(-0.09, 0.33)	0.261	0.20(-0.15, 0.55)	0.267	0.14(-0.03, 0.31)	0.098
Female-to-male (Sex) ratio	-0.16(-0.49, 0.17)	0.335	0.04(-0.17, 0.25)	0.698	0.12(-0.13, 0.36)	0.351	-0.08(-0.48,0.33)	0.711	0.07(-0.12, 0.27)	0.476
Household size	0.03(-0.05, 0.10)	0.518	0.01(-0.38, 0.61)	0.654	0.11(0.05, 0.17)	0.000	-0.03(-0.13,0.07)	0.542	0.02(-0.02, 0.07)	0.324
Literacy ratio	-0.01(-0.48, 0.47	0.982	-0.10(-0.41,0.20)	0.516	-0.09(-0.44, 0.25)	0.597	-0.11(-0.70,0.47)	0.701	-0.10(-0.38,0.18)	0.489
Farm diversity	-0.06(-0.33, 0.20)	0.638	0.15(-0.02, 0.32)	0.077	0.34(0.15, 0.53)	0.001	0.53(0.21, 0.86)	0.001	0.23(0.07, 0.38)	0.004
Crop diversity	-0.09(-0.47, 0.28)	0.613	0.14(-0.09, 0.38)	0.249	0.35(0.07, 0.62)	0.013	0.71(0.25, 1.16)	0.002	0.31(0.10, 0.53)	0.005
Animal Diversity	-0.08(-070, 0.54)	0.794	0.45(0.05,0.85)	0.027	0.74(0.29, 1.20)	0.001	0.82(0.05, 1.58)	0.036	0.36(-0.00, 0.73)	0.050
**Household food security status**										
Food secured	Ref		Ref		Ref		Ref		Ref	
Mildly food insecure	0.46(-0.74,1.65)	0.453	-0.45(-1.20,0.31)	0.243	-1.18(-2.05, -0.31)	0.008	-0.99(-2.46,0.48)	0.186	-0.72(-1.42, -0.02)	0.043
Moderately/ Severely food insecure	-1.40(-0.66, 1.46)	0.463	-1.65(-2.33,0.99)	0.000	-1.93(-2.70, -1.15)	0.000	-0.69(-2.00,0.61)	0.298	-0.45(-1.08,0.17)	0.153
**Quintiles of wealth index**										
Quintile 1	Ref		Ref		Ref		Ref		Ref	
Quintile 2	0.32(-0.94, 1.59)	0.618	0.47(-0.34, 1.29)	0.254	0.83(-0.09, 1.76)	0.078	0.99(-0.57,2.55)	0.213	0.55(-0.19,1.29)	0.148
Quintile 3	-0.13(-1.39, 1.13)	0.838	0.55(-0.26, 1.36)	0.182	0.44(-0.48, 1.36)	0.347	1.43(-0.12, 2.98)	0.071	0.100(0.26,1.73)	0.008
Quintile 4	0.52(-0.77, 1.81)	0.429	0.60(-0.23, 1.43)	0.155	1.36(0.42, 2.31)	0.005	1.35(-0.24, 2.95)	0.096	0.73(-0.02, 1.49)	0.058
Quintile 5	0.99(-0.31, 2.29)	0.134	0.94(0.10, 1.77)	0.028	1.79(0.83, 2.73)	0.000	1.41(-0.18, 3.01)	0.083	1.14(0.38, 1.90)	0.003

Table S5: Factors Associated with Frequency of Consumption of Fruits and Vegetables among the Adolescent Girls: Bivariate Linear Mixed-Effect Model Analysis

Variables	Fruits		Vegetables	
	**Estimate (95 % C.I)**	**P-value**	**Estimate (95 % C.I)**	**P-value**
Age of girls (years)	0.08(-0.13, 0.28)	0.461	0.07(-0.29, 0.44)	0.700
**Ethnicity**				
Dagomba	-0.74(-3.61, 2.12)	0.609	-0.62(-3.61, 2.36)	0.683
Konkomba	-1.23(-4.12, 1.67)	0.406	-0.57(-3.59, 2.44)	0.709
Other	Ref		Ref	
**Religion**				
Muslim	-1.06(-4.23, 2.11)	0.513	-4.23(-9.96, 1.50)	0.148
Christian	-1.63(-4.83, 1.57)	0.318	-3.83(-9.61, 1.95)	0.194
Other	Ref		Ref	
**Menarche Status**				
Pre-menarche	0.08(-0.87, 1.03)	0.873	-0.41(-2.13, 1.31)	0.641
Post-menarche	Ref		Ref	
**Maternal literacy**				
Non-literate	-1.22(-2.66, 0.21)	0.095	0.65(-1.94, 3.25)	0.621
Literate	Ref		Ref	
Maternal decision index	-0.01(-0.28, 0.27)	0.962	-0.45(-0.94, 0.04)	0.074
Female-to-male (Sex) ratio	-0.14(-0.46, 0.17)	0.382	-0.20(-0.77, 0.37)	0.491
Household size	0.03(-0.04, 0.10)	0.369	0.01(-0.13, 0.14)	0.902
Literacy ratio	0.07(-0.38. 0.53)	0.754	-0.08(-0.91, 0.74)	0.844
Farm diversity	0.19(-0.06, 0.45)	0.140	-0.33(-0.79, 0.13)	0.158
Crop diversity	0.19(-0.16, 0.55)	0.280	-0.40(-1.05, 0.25)	0.225
Animal Diversity	0.49(-0.10, 1.08)	0.107	-0.69(-1.76, 0.39)	0.211
**Household food security status**				
Food secured	Ref		Ref	
Mildly food insecure	-2.02(-3.16, -0.89)	0.000	2.93(0.87, 4.99)	0.005
Moderately/ Severely food insecure	-2.40(-3.41, -1.39)	0.000	3.16(1.33, 4.99)	0.001
**Quintiles of wealth index**				
Quintile 1	Ref		Ref	
Quintile 2	0.24(-0.97, 1.46)	0.694	0.43(-1.77, 2.64)	0.698
Quintile 3	-0.19(-1.40, 1.02)	0.758	-0.02(-2.20, 2.17)	0.989
Quintile 4	0.50(-0.74, 1.75)	0.423	0.54(-1.70, 2.79)	0.635
Quintile 5	0.29(-0.96, 1.53)	0.653	1.63(-0.62, 3.88)	0.157
